# Differences in colorectal cancer awareness between screening eligible vs. ineligible Palestinians: a national cross-sectional study

**DOI:** 10.1093/eurpub/ckae083

**Published:** 2024-05-14

**Authors:** Mohamedraed Elshami, Maram Albandak, Mohammed Alser, Ibrahim Al-Slaibi, Mohammed Ayyad, Mohammad F Dwikat, Shoruq A Naji, Balqees M Mohamad, Wejdan S Isleem, Adela Shurrab, Bashar Yaghi, Yahya Ayyash Qabaja, Fatma K Hamdan, Raneen R Sweity, Remah T Jneed, Khayria A Assaf, Mohammed M Hmaid, Iyas I Awwad, Belal K Alhabil, Marah N Alarda, Amani S Alsattari, Moumen S Aboyousef, Omar A Aljbour, Rinad AlSharif, Christy T Giacaman, Ali Y Alnaga, Ranin M Abu Nemer, Nada M Almadhoun, Sondos M Skaik, Shurouq I Albarqi, Nasser Abu-El-Noor, Bettina Bottcher

**Affiliations:** Division of Surgical Oncology, Department of Surgery, University Hospitals Cleveland Medical Center, Cleveland, OH, USA; Ministry of Health, Gaza, Palestine; Faculty of Medicine, Al-Quds University, Jerusalem, Palestine; The United Nations Relief and Works Agency for Palestine Refugees in the Near East (UNRWA), Gaza, Palestine; Almakassed Hospital, Jerusalem, Palestine; Faculty of Medicine, Al-Quds University, Jerusalem, Palestine; Faculty of Medicine, An-Najah National University, Nablus, Palestine; Faculty of Pharmacy, Al-Azhar University of Gaza, Gaza, Palestine; Doctors Without Borders (Médecins Sans Frontières), Hebron, Palestine; Faculty of Medicine, Islamic University of Gaza, Gaza, Palestine; Palestine Medical Complex, Khan Younis, Palestine; Faculty of Medicine, Al-Quds University, Jerusalem, Palestine; Faculty of Medicine, Al-Quds University, Jerusalem, Palestine; Faculty of Medicine, Al-Quds University, Jerusalem, Palestine; Faculty of Medicine, Al-Quds University, Jerusalem, Palestine; Faculty of Medicine, Islamic University of Gaza, Gaza, Palestine; Faculty of Medicine, An-Najah National University, Nablus, Palestine; Faculty of Medicine, Islamic University of Gaza, Gaza, Palestine; Faculty of Medicine, Al-Quds University, Jerusalem, Palestine; Ministry of Health, Gaza, Palestine; Faculty of Dentistry, Arab American University, Jenin, Palestine; Faculty of Medicine, Islamic University of Gaza, Gaza, Palestine; Faculty of Medicine, Islamic University of Gaza, Gaza, Palestine; Faculty of Medicine, Islamic University of Gaza, Gaza, Palestine; Faculty of Medicine, Al-Quds University, Jerusalem, Palestine; Augusta Victoria Hospital, Jerusalem, Palestine; Faculty of Medicine, Islamic University of Gaza, Gaza, Palestine; Faculty of Allied Medical Sciences, Arab American University, Jenin, Palestine; Faculty of Medicine, Al-Azhar University, Gaza, Palestine; Faculty of Medicine, Al-Azhar University, Gaza, Palestine; Faculty of Pharmacy, Al-Azhar University of Gaza, Gaza, Palestine; Faculty of Nursing, Islamic University of Gaza, Gaza, Palestine; Faculty of Medicine, Islamic University of Gaza, Gaza, Palestine

## Abstract

**Background:**

This study aimed to compare colorectal cancer (CRC) awareness between screening-eligible and ineligible individuals in Palestine.

**Methods:**

Convenience sampling was utilized to recruit Palestinian adults from diverse settings, including hospitals, primary healthcare centers and public spaces across 11 governorates. The evaluation of CRC awareness in terms of signs/symptoms, risk factors and causation myths was conducted using Arabic-translated, modified versions of the validated instruments, the Bowel Cancer Awareness Measure and the Cancer Awareness Measure-Mythical Causes Scale.

**Results:**

The final analysis included 2698 participants, with 2158 (80.9%) eligible for CRC screening and 540 (19.1%) ineligible for it. The most recognized CRC sign/symptom was ‘lump in the abdomen’ in both screening-eligible (n = 386, 71.5%) and ineligible (n = 1582, 73.3%) groups. ‘Lack of physical activity’ was the most recognized risk factor in both groups (eligible: n = 451, 83.5%; ineligible: n = 1766, 81.8%). The most reported causation myth in both groups was ‘having a physical trauma’ (eligible: n = 340, 63.0%; ineligible: n = 1353, 62.7%). In the screening-eligible group, only 210 participants (38.9%) demonstrated high awareness of CRC signs/symptoms, 213 participants (39.4%) showed high awareness of CRC risk factors and only 46 participants (8.5%) displayed high awareness of CRC causation myths. There were no significant associations between being eligible for screening colonoscopy and the awareness levels of CRC signs/symptoms, risk factors and causation myths.

**Conclusion:**

Overall, awareness levels of CRC signs/symptoms, risk factors and causation myths were notably low among screening-eligible participants. There were no differences in awareness levels between individuals eligible for colonoscopy and those who were not.

## Introduction

About 70% of cancer-related deaths occur in low and middle-income countries because of barriers in accessing timely diagnostic and therapeutic interventions.^1^ Additionally, a significant proportion of cancer cases in these regions are identified in advanced stages, primarily due to inadequate screening protocols and a lack of awareness.^2^ Colorectal cancer (CRC) is the third most frequently diagnosed malignancy globally.^3^ By 2024, the numbers of CRC are anticipated to rise with 3.2 million new cases and 1.6 million deaths; a 63% increase from 2020.^4^ In Palestine, CRC constitutes the second most common cancer following breast cancer, with incidence rates reported at 15.3 per 100 000 in the general population of the West Bank and 10.2 per 100 000 in the Gaza Strip.^5^

A substantial number of CRC cases can be prevented, or the disease progression mitigated, by adhering to recommended screening protocols, particularly through colonoscopy.^6^^,^^7^ However, there is a pronounced underutilization of CRC screening in comparison to screenings for other types of cancer.^8^ The American Cancer Society recommends starting CRC screening for individuals with an average risk of developing the disease at the age of 45 years.^9^ To date, Palestine lacks a national screening program for CRC. Qumseya et al.^10^ reported that based on preliminary data and discussions with healthcare professionals in the West Bank, there is an anticipated markedly low rate of CRC screening in the region, expected to be less than 15%, in comparison to 64% in the USA.^11^ Considering the current lack of CRC awareness initiatives and regular screening programs in Palestine, it is imperative to implement an effective strategy focused on identifying high-risk groups based on personal and family history. This strategy should involve promoting awareness campaigns and consolidating physician efforts to streamline referral processes, ensuring prompt evaluation of patients presenting with possible CRC symptoms.^12^

Despite the persistent low levels of awareness in Palestine regarding CRC signs/symptoms, risk factors and misconceptions about its causation (e.g. eating burnt food can cause CRC), as evidenced by previous studies,^13–15^ there remains an unexplored area in the existing literature concerning variations in awareness between individuals eligible for CRC screening and those who are not. Understanding the existing knowledge regarding CRC among Palestinians who are eligible for screening becomes even more critical as possible means for early detection and prevention.^13–16^ Therefore, this national study aimed to compare awareness levels of CRC signs/symptoms, risk factors and causation myths between screening-eligible and ineligible Palestinians.

## Methods

### Study design and population

This nationwide cross-sectional study was conducted from July 2019 to March 2020. The study targeted Palestinian adults aged 18 years and above residing in the West Bank, Jerusalem, and the Gaza Strip, constituting approximately 62.2% of the total Palestinian population estimated at 5 million.^5^ Inclusion criteria comprised Palestinian adults visiting designated data collection sites. Conversely, exclusion criteria encompassed visiting oncology departments during the data collection period, engaging in health-related studies or professions, citizenship other than Palestinian, having a family history of cancer, history of colorectal or gastrointestinal disease, and being unable to complete the questionnaire.

### Sampling methods

In Palestine, government hospitals and primary healthcare centers deliver services with minimal or no cost, rendering them the most accessible and commonly used healthcare providers for the majority of patients.^17^ Palestine consists of 16 governorates, five in the Gaza Strip and 11 in the West Bank and Jerusalem.^18^ In consistence with previous reporting,^13^^,^^14^^,^^16^ convenience sampling was utilized to recruit participants from governmental hospitals, primary healthcare centers and public spaces across 11 governorates in Palestine, four located in the Gaza Strip and seven in the West Bank and Jerusalem.

### Data collection and measurement tool

The study employed two validated questionnaires, the Bowel Cancer Awareness Measure (BoCAM) and the Cancer Awareness Measure-Mythical Causes Scale (CAM-MYCS).^19^^,^^20^ Modified versions of the BoCAM and CAM-MYCS were used after a back-to-back translation process, which involved converting the questionnaires from English to Arabic by two bilingual healthcare professionals. Subsequently, two additional healthcare professionals, proficient in both languages and experienced in clinical research and survey design, independently back-translated the questionnaire into English. Moreover, five independent experts in public health, gastroenterology and coloproctology evaluated the translated questionnaire to ensure both content validity and translation accuracy. A pilot study (*n* = 25) was conducted to assess the clarity of the questions in the Arabic version of the questionnaire, and its responses were excluded from the final analysis. The internal consistency of the questionnaire was then assessed using Cronbach’s alpha, yielding an acceptable value of 0.81.

The questionnaire included four sections. The first section focused on collecting sociodemographic information. In the second section, participants’ awareness of 12 CRC signs and symptoms was evaluated based on a 5-point Likert scale, ranging from 1 (strongly disagree) to 5 (strongly agree). The third section explored participants’ awareness of 11 CRC risk factors, using the same 5-point Likert scale. Among the 12 CRC signs and symptoms, nine were retained from the original BoCAM.^19^ Additionally, three symptoms—‘feeling persistently full,’ ‘unexplained loss of appetite,’ and ‘unexplained generalized fatigue’—were introduced based on alternative versions of the Cancer Awareness Measure.^21^^,^^22^ Similarly, 10 risk factors were adapted from the original BoCAM,^19^ and an additional risk factor, ‘smoking cigarettes,’ was included due to its high prevalence in the Palestinian population.^23^ In the fourth section of the questionnaire, participants’ ability to recognize 13 myths pertaining to CRC causation myths was evaluated. Twelve of these myths were adapted from the original CAM-MYCS instrument.^20^ Additionally, an extra myth related to ‘eating burnt food’ was included due to its existence within the Palestinian community.^24^

Data were collected using Kobo Toolbox, a secure and user-friendly tool accessible via smartphones.^25^ Data collectors had a medical background and received comprehensive training to help participants complete the questionnaire through face-to-face interviews utilizing the Kobo Toolbox platform.

### Exposure and outcome

Previous studies from our group in Palestine demonstrated a suboptimal awareness of CRC in the general population.^13–15^ However, no studies have examined CRC awareness in screening-eligible individuals and compared it to that in screening-ineligible individuals. Therefore, the exposure of interest in this follow-up study was eligibility to undergo CRC screening, defined as aging 45–75 years while having an average-risk of developing CRC (i.e. no family history of cancer or personal history of colorectal disease). The primary outcome was the proportion of CRC screening-eligible participants displaying high awareness of CRC signs/symptoms, risk factors and causation myths.

### Statistical analysis

Interquartile range (IQR) and median were used to summarize continuous variables with non-normal distribution, and the Kruskal–Wallis test was used to make baseline comparisons between participants who were eligible for screening and those who were not. Meanwhile, frequencies and percentages were used to summarize categorical variables, and Pearson’s chi-square test was used to perform baseline comparisons between screening-eligible and ineligible participants.

CRC signs/symptoms were categorized into three distinct groups based on their characteristics: those indicative of a mass or blood presence in stools, those of a non-specific nature, and other signs and symptoms related to the gastrointestinal tract. Likewise, CRC risk factors were categorized into two group: modifiable and non-modifiable factors. The identification of CRC signs/symptoms and risk factors was assessed using a 5-point Likert scale. Correct responses were defined as ‘strongly agree’ or ‘agree,’ while ‘strongly disagree,’ ‘disagree,’ or ‘not sure’ were deemed incorrect answers. Categorization of myths pertaining to the causation of CRC involved classifying them into two groups: those related to food and those unrelated to food. Answers with ‘disagree’ or ‘strongly disagree’ were considered correct, while all other responses were considered incorrect.

Prompt recognition of CRC signs/symptoms, risk factors and causation myths was described using frequencies and percentages, with comparisons between screening-eligible and ineligible participants performed using Pearson’s chi-square test. Following this, a multivariable logistic regression analysis was performed to examine the association between eligibility to undergo CRC screening and the recognition of CRC signs/symptoms, risk factors and causation myths. The multivariable model was adjusted for various covariates, including age, gender, education, occupation, monthly income, place of residence, marital status, presence of a chronic disease, knowing someone with cancer and the site of data collection. The selection of these covariates was determined *a priori* based on previous studies.^13–16^

The assessment of awareness levels regarding CRC signs/symptoms, risk factors and causation myths employed a scoring system that is consistent with previous studies.^13–16^ One point was assigned for each correctly recognized item, contributing to the calculation of total awareness scores for each domain. These scores were subsequently categorized into tertiles: the lower two tertiles representing ‘low’ awareness and the upper tertile representing ‘high’ awareness. Descriptive statistics, including frequencies and percentages, were employed to summarize awareness levels, and comparisons between CRC screening-eligible and ineligible participants were conducted using Pearson’s chi-square test. Subsequently, a multivariable logistic regression analysis was conducted to examine the association between eligibility to undergo CRC screening and the demonstration of high awareness within each domain, utilizing the same aforementioned multivariable model.

Missing data were hypothesized to be missed completely at random and thus, complete case analysis was utilized to handle them. Data were analyzed using Stata software version 17.0 (StataCorp, College Station, TX, USA).

### Ethical approval and consent to participate

Ethical approval was obtained from the Research Ethics Committee at the Islamic University of Gaza, the Human Resources Development department at the Palestinian Ministry of Health and the Helsinki Committee in the Gaza Strip prior to data collection. The study adhered to local guidelines and regulations. Participants were provided with an overview of the study’s objectives, emphasizing the voluntary nature of their participation. Written informed consent was obtained prior to participation in the study.

## Results

### Participant characteristics

The final analysis included 2698 participants, with 2158 (80.9%) eligible for screening colonoscopy and 540 (19.1%) ineligible. The median age [IQR] for the CRC screening-eligible group was 52.0 [47.0, 57.0] years, while the ineligible group had a median age [IQR] of 27.0 [23.0, 34.0] years. Participants in the CRC screening-eligible group were older, reported a higher monthly income but lower education, and had more chronic diseases. More detailed information about participant characteristics can be found in Supplementary table S1.

### Recognition of CRC signs/symptoms

The most frequently recognized CRC sign/symptom in the CRC screening-eligible group was ‘lump in the abdomen’ (*n* = 386, 71.5%), followed by ‘blood in the stools’ (*n* = 361, 66.9%) (table 1). Similarly, ‘lump in the abdomen’ was the most identified sign/symptom in the screening-ineligible group (*n* = 1582, 73.3%), followed by blood in the stools (*n* = 1443, 66.9%) and ‘unexplained weight loss’ (*n* = 1443, 66.9%). Conversely, the least frequently recognized sign/symptom in both groups was ‘pain in the back passage’ (screening-eligible group: *n* = 264, 48.9% vs. screening-ineligible group: *n* = 961, 44.5%). Participants in the screening-eligible group were more likely to recognize ‘anemia’ (OR = 1.34, 95% CI: 1.06–1.71) and ‘change in bowel habits’ (OR = 1.36, 95% CI: 1.07–1.71) as possible CRC signs/symptoms than participants in the screening-ineligible group.

**Table 1:** Recognition of colorectal cancer symptoms, stratified by eligibility to undergo colorectal cancer screening

Category of sign/symptom	Sign/symptom	Screening-ineligible	Screening-eligible	OR (95% CI)	*P*-value
		(*n* = 2158)	(*n* = 540)		
**Signs/symptoms with a mass or blood**	Lump in the abdomen	1582 (73.3)	386 (71.5)	0.85 (0.66–1.10)	0.22
	Blood in the stools	1443 (66.9)	361 (66.9)	1.05 (0.82–1.34)	0.69
	Bleeding from back passage	1301 (60.3)	323 (59.8)	0.90 (0.71–1.14)	0.37
**Signs/symptoms of a non-specific nature**	Unexplained weight loss	1443 (66.9)	371 (68.7)	0.99 (0.77–1.27)	0.95
	Unexplained generalized fatigue	1400 (64.9)	353 (65.4)	0.98 (0.77–1.25)	0.87
	Unexplained loss of appetite	1252 (58.0)	304 (56.3)	0.91 (0.72–1.14)	0.41
	Anemia	1195 (55.4)	350 (64.8)	1.34 (1.06–1.71)	0.016
**Other gastrointestinal signs/symptoms**	Feeling persistently full	1198 (55.5)	307 (56.9)	0.99 (0.78–1.25)	0.92
	Change in bowel habits	1154 (53.5)	327 (60.6)	1.36 (1.07–1.71)	0.011
	Persistent pain in the abdomen	1241 (57.5)	294 (54.4)	0.84 (0.66–1.06)	0.14
	Bowel does not completely empty after using the lavatory	1031 (47.8)	287 (53.1)	1.17 (0.93–1.47)	0.19
	Pain in the back passage	961 (44.5)	264 (48.9)	1.14 (0.90–1.43)	0.27

*n*, number of participants; OR, odds ratio; CI, confidence interval.

### Recognition of CRC risk factors

‘Lack of physical activity’ was the most identified modifiable risk factor for CRC in both screening-eligible (*n* = 451, 83.5%) and ineligible (*n* = 1766, 81.8%) groups (table 2). In contrast, the modifiable risk factor with the least recognition in both groups was ‘having a diet low in fiber’ (screening-eligible group: *n* = 251, 46.5% vs. screening-ineligible group: *n* = 939, 43.5%).

**Table 2:** Recognition of colorectal cancer risk factors, stratified by eligibility to undergo colorectal cancer screening

Risk factor	Screening-ineligible	Screening-eligible	OR (95% CI)	*P*-value
	(*n* = 2158)	(*n* = 540)		
Modifiable risk factors				
Not doing 30 min of moderate physical activity 5 times a week	1766 (81.8)	451 (83.5)	0.85 (0.62–1.15)	0.29
Smoking cigarettes	1610 (74.6)	408 (75.6)	1.00 (0.77–1.33)	0.95
Drinking alcohol	1544 (71.5)	384 (71.1)	1.00 (0.77–1.30)	0.98
Not eating five portions of fruits & vegetables a day	1526 (70.7)	400 (74.1)	1.17 (0.90–1.51)	0.24
Being overweight	1389 (64.4)	369 (68.3)	1.17 (0.92–1.50)	0.20
Eating red meat once a day or more	1060 (49.1)	285 (52.8)	1.18 (0.93–1.48)	0.17
Having a diet low in fiber (e.g. fruits and vegetables)	939 (43.5)	251 (46.5)	1.14 (0.90–1.44)	0.28
Non–modifiable risk factors				
Having a bowel disease (e.g. inflammatory bowel disease)	1494 (69.2)	374 (69.3)	0.97 (0.76–1.25)	0.82
Having a close relative with bowel cancer	1224 (56.7)	278 (51.5)	0.72 (0.57–0.91)	0.006
Being over 70 years old	950 (44.0)	260 (48.1)	1.26 (1.00–1.58)	0.054
Having diabetes	733 (34.0)	179 (33.1)	0.97 (0.76–1.24)	0.79

*n*, number of participants; OR, odds ratio; CI, confidence interval.

‘Having a bowel disease’ was the most identified non-modifiable risk factor among both screening-eligible (*n* = 374, 69.3%) and ineligible (*n* = 1494, 69.2%) groups. Conversely, the least recognized non-modifiable risk factor in both groups was ‘having diabetes’ (screening-eligible group: *n* = 179, 33.1% vs. screening-ineligible group: *n* = 733, 34.0%). Screening-eligible participants were less likely to recognize ‘having a close relative with bowel cancer’ (OR= 0.72, 95% CI: 0.57–0.91) as a risk factor for CRC than screening-ineligible participants.

### Recognition of CRC causation myths

Regarding public beliefs of the mythical causes of CRC, ‘eating burnt food’ was the most reported food-related myth in both screening-eligible (*n* = 154, 28.5%) and ineligible (*n* = 559, 25.9%) groups (table 3). Conversely, ‘eating food containing additives’ was the least reported food-related myth in both screening-eligible (*n* = 81, 15.0%) and ineligible (*n* = 257, 11.9%) groups.

**Table 3:** Recognition of colorectal cancer causation myths, stratified by eligibility to undergo colorectal cancer screening

Causation myth	Screening-ineligible	Screening-eligible	OR (95% CI)	*P*-value
	(*n* = 2158)	(*n* = 540)		
Food-related				
Eating burnt food (e.g. bread or barbeque)	559 (25.9)	154 (28.5)	0.96 (0.74–1.23)	0.73
Drinking from plastic bottles	555 (25.7)	139 (25.7)	1.00 (0.77–1.32)	0.97
Using microwave ovens	509 (23.6)	131 (24.3)	0.95 (0.72–1.24)	0.69
Eating food containing artificial sweeteners (e.g. saccharine)	482 (22.3)	138 (25.6)	1.21 (0.92–1.58)	0.18
Eating genetically modified food (e.g. hybrid vegetables)	355 (16.5)	108 (20.0)	1.27 (0.95–1.70)	0.11
Eating food containing additives	257 (11.9)	81 (15.0)	1.02 (0.73–1.43)	0.91
Food-unrelated				
Having a physical trauma	1353 (62.7)	340 (63.0)	1.02 (0.73–1.43)	0.91
Using aerosol containers	1016 (47.1)	245 (45.4)	0.81 (0.64–1.03)	0.08
Using cleaning products	929 (43.0)	231 (42.8)	0.84 (0.67–1.07)	0.15
Using mobile phones	882 (40.9)	192 (35.6)	0.91 (0.72–1.16)	0.46
Living near power lines	764 (35.4)	199 (36.9)	1.14 (0.90–1.45)	0.28
Exposure to electromagnetic frequencies (e.g. Wi-Fi and Radio/TV frequencies)	656 (30.4)	193 (35.7)	1.03 (0.80–1.31)	0.83
Feeling stressed	725 (33.6)	186 (34.4)	0.84 (0.66–1.07)	0.16

*n*, number of participants; OR, odds ratio; CI, confidence interval.

‘Having a physical trauma’ was the most recognized food-unrelated myth in both groups (screening-eligible: *n* = 340, 63.0% vs. screening-ineligible: *n* = 1353, 62.7%). In contrast, the least recognized food-unrelated myth was ‘feeling stressed’ in both groups (screening-eligible group: *n* = 186, 34.4% vs. screening-ineligible group *n* = 725, 33.6%).

### CRC awareness and eligibility for CRC screening

A total of 210 participants (38.9%) in the screening-eligible group demonstrated high awareness of CRC signs/symptoms, while 777 participants in the screening-ineligible group (36.0%) showed similar awareness (table 4). Similarly, the majority in both the screening-eligible and ineligible groups did not exhibit a high awareness of CRC risk factors, with only 213 (39.4%) and 814 (37.7%) displaying it, respectively. Furthermore, most participants in both groups did not demonstrate a high awareness of CRC causation myths, with only 46 (8.5%) of the screening-eligible group and 120 (5.6%) of the screening-ineligible group (*n* = 120) displaying high awareness. There were no significant associations between being eligible for CRC screening and the awareness level of CRC signs/symptoms, risk factors and causation myths.

**Table 4:** Association between being eligible to undergo colorectal cancer screening and displaying high awareness of each of colorectal cancer symptoms, risk factors and causation myths

Outcome	Screening-ineligible	Screening-eligible	OR (95% CI)	*P*-value
	(*n* = 2158)	(*n* = 540)		
**Awareness of CRC symptoms**				
Low	1381 (64.0)	330 (61.1)	Ref	Ref
High	777 (36.0)	210 (38.9)	1.04 (0.82–1.32)	0.73
**Awareness of CRC risk factors**				
Low	1344 (62.3)	327 (60.6)	Ref	Ref
High	814 (37.7)	213 (39.4)	1.08 (0.85–1.37)	0.51
**Awareness of CRC causation myths**				
Low	2038 (94.4)	494 (91.5)	Ref	Ref
High	120 (5.6)	46 (8.5)	1.28 (0.82–2.00)	0.27

*n*, number of participants; OR, odds ratio; CI, confidence interval; CRC, colorectal cancer.

## Discussion

In this study, the awareness levels of CRC signs/symptoms, risk factors and causation myths were assessed and compared between screening-eligible and ineligible participants in Palestine. More than one-third of participants who were eligible to CRC screening demonstrated high awareness of CRC signs/symptoms and risk factors. However, a minority of them (<5%) showed high awareness of CRC causation myths. There were no associated differences in the awareness level of CRC signs/symptoms, risk factors and causation myths between CRC screening-eligible and ineligible participants.

### Awareness of CRC signs/symptoms in screening eligible vs. ineligible groups

Multiple studies have indicated a positive association between the awareness of CRC symptoms and risk factors and the adoption of CRC screening measures.^26–28^ ‘Lump in the abdomen’ was the most recognized CRC sign/symptom in both screening eligible and ineligible groups, consistent with previous studies conducted in Palestine.^13^^,^^16^ Similarly, a study from Qatar reported that ‘lump in the abdomen’ was the most recognized CRC symptom.^29^ Understanding these shared patterns of CRC awareness is crucial for overcoming barriers and establishing effective CRC screening programs. A previous study from Malaysia, which utilized a culturally adapted mass media campaign to enhance CRC awareness and reduce the rates of late diagnosis, reported notable improvements in symptom awareness among the Malaysian population.^30^ This demonstrates the potential effectiveness of interventions that incorporate cultural and social considerations. Therefore, integrating such factors into behavioral interventions may be instrumental in promoting CRC screening practices.

On the other hand, the least frequently recognized sign/symptom in both groups was ‘pain in the back passage’. A previous study from Palestine has indicated that symptoms of CRC with blood or mass tend to prompt seeking earlier medical advice.^16^ In addition, participants demonstrating high awareness of CRC were more inclined to seek medical advice within a week.^16^ Hence, the reduction of delays in seeking medical care is contingent on individuals having good awareness of CRC signs and symptoms, including the less specific symptoms that are less likely to be recognized as warning signs. Therefore, future educational interventions should focus more on improving the awareness of those less specific CRC symptoms, which may encourage individuals to promptly seek medical advice for seemingly less alarming symptoms to minimize delays in care and improve overall outcomes.

Interestingly, screening-eligible participants were more likely to recognize ‘anemia’ and ‘change in bowel habits’ as CRC symptoms. This phenomenon may be attributable to differences in health literacy between both groups, suggesting a predominant emphasis on more overt symptoms in CRC screening education. This emphasis could potentially contribute to the reduced awareness of subtler indicators like anemia or changes in bowel habits among screening-ineligible participants, especially as they are less likely to interfere with day-to-day activities.

### Awareness of CRC risk factors in screening eligible vs. ineligible groups

In both screening eligible and ineligible groups, the most commonly identified modifiable risk factor for CRC was ‘lack of physical activity,’ while ‘having a bowel disease’ stood out as the most recognized non-modifiable risk factor, consistent with previous studies in Palestine.^14^ The increased awareness of ‘lack of physical activity’ may be attributed to various campaigns promoting an active lifestyle in Palestine.^31^ Additionally, the increased recognition of ‘having a bowel disease’ as a risk factor for CRC could be influenced by the rising incidence of inflammatory bowel disease in the Middle East, which has been linked to westernization of diet.^32^

On the contrary, the least recognized non-modifiable risk factor was ‘having diabetes’. This finding aligns with a study in the UK, where only 25.8% of participants were aware of diabetes as a risk factor for CRC.^19^ Similar observations were noted in urban and rural populations of Klang Valley.^33^ This underscores the importance of healthcare professionals actively educating the public about the association between diabetes and the risk of developing CRC.

Previous studies from Norway, the UK and Spain consistently demonstrated a robust link between knowledge of CRC risk factors and the intent to participate in forthcoming CRC screening programs.^34–36^ Hence, there is a reasonable expectation that increasing public awareness regarding CRC prevention will reduce negative public perceptions of cancer and contribute to increased participation in CRC screening. In fact, our group recently has found that having high awareness of CRC symptoms and risk factors was associated with favorable attitudes toward screening colonoscopy.^37^ This highlights the importance of tailored public health campaigns and advocacy for CRC screening, ultimately promoting early CRC detection and intervention.

### Awareness of CRC causation myths in in screening eligible vs. ineligible groups

Regarding public beliefs of the mythical causes of CRC, both screening eligible and ineligible groups commonly identified ‘eating burnt food’ as a food-related myth and ‘having a physical trauma’ as a food-unrelated myth. These findings contrast with a study in the UK that investigated the prevalence of beliefs regarding both actual and mythical causes of cancer. The study indicated that around one-third of participants linked cancer risk to factors such as stress, genetically modified food, food additives and non-ionizing electromagnetic frequencies.^38^ Beliefs in such myths may correlate with increased anxiety, particularly when the perceived risk is beyond individual control.^39^^,^^40^ Fostering more accurate causal beliefs could potentially alleviate cancer-related fear and empower individuals to take proactive measures in reducing their risk, especially those who are eligible to undergo CRC screening.

### Lack of association between eligibility to undergo CRC screening and displaying high CRC awareness

Our study revealed no significant associations between eligibility for CRC screening and awareness levels of CRC signs/symptoms, risk factors and causation myths. This can be attributed to the overall low awareness levels in the Palestinian population,^13–16^ highlighting a persistent knowledge gap. Furthermore, it suggests that screening eligibility alone may not determine or properly reflect individuals’ awareness of CRC. Meeting the criteria for CRC screening, which typically involves age considerations and other risk factors, may not necessarily correlate with a higher level of knowledge about the disease. Consequently, targeted informational campaigns are essential to increase awareness and dispel common misconceptions, which may also encourage individuals to undergo CRC screening when available.

### Future directions

Enhancing public awareness of CRC is critical for increasing participation in CRC screening initiatives. This could be accomplished by implementing strategic and culturally adapted educational campaigns coupled with online social platforms to enhance outreach, education and engagement in CRC screening efforts. These efforts should target both screening-eligible and ineligible populations to increase overall CRC awareness in Palestine and establish the foundation for a national screening program. While information about cancer prevention may not directly induce behavioral change, it serves as a facilitator and has the potential to motivate individuals to adopt healthier lifestyles. Additionally, fostering accurate causal beliefs within the population has the potential to reduce cancer-related anxiety and empower individuals to actively undertake proactive measures to mitigate their associated risk.

### Limitations

This study is subject to certain limitations. Convenience sampling may not ensure a fully representative sample, impacting the generalizability of findings. Despite this, the study’s substantial sample size, high response rate and broad geographic coverage may have mitigated this limitation. The exclusion of individuals from oncology departments and those with medical backgrounds may have lowered the numbers of participants with presumably good CRC awareness. However, this aligns with the primary focus of the study on specific groups of interest. The study also assessed participants’ perceived knowledge, and this may differ in the presence of actual CRC diagnosis. Lastly, we attempted to account for differences in the groups of interest (screening-eligible vs. screening-ineligible) by including several known sociodemographic variables in the multivariable analyses. However, we could not adjust for unknown characteristics that may differ between both groups.

## Supplementary Material

ckae083_Supplementary_Data

## Data Availability

The datasets used and analyzed during the current study are available from the corresponding author on reasonable request. Key points Both colorectal cancer (CRC) screening-eligible and ineligible participants in Palestine demonstrated low awareness levels of CRC signs/symptoms, risk factors and causation myths. There were no associations between eligibility for CRC screening and awareness levels of CRC signs/symptoms, risk factors and causation myths. Strategic and culturally adapted campaigns are crucial for increasing overall CRC awareness, especially targeting both screening-eligible and ineligible populations, to lay the foundation for an effective national screening program in Palestine.

## References

[1] American Cancer Society. The Global Cancer Burden. Available at: https://bit.ly/3NUbCDk (12 April 2024, date last accessed).

[2] World Health Organization. Cancer. Available at: https://bit.ly/3NJSvw3 (12 April 2024, date last accessed).

[3] Navarro M , Nicolas A, Ferrandez A, Lanas A. Colorectal cancer population screening programs worldwide in 2016: an update. World J Gastroenterol 2017;23:3632–42. 28611516 10.3748/wjg.v23.i20.3632 PMC5449420

[4] Morgan E , Arnold M, Gini A, et al Global burden of colorectal cancer in 2020 and 2040: incidence and mortality estimates from GLOBOCAN. Gut 2023;72:338–44. 36604116 10.1136/gutjnl-2022-327736

[5] Palestinian Ministry of Health. Annexes to the Annual Health Report of Palestine 2022. Available at: https://bit.ly/3ZR5v7G (12 April 2024, date last accessed).

[6] U.S. Preventive Services Task Force. Screening for colorectal cancer: U.S. Preventive Services Task Force recommendation statement. Ann Intern Med 2008;149:627–37. 18838716 10.7326/0003-4819-149-9-200811040-00243

[7] Brenner H , Chang-Claude J, Seiler CM, et al Protection from colorectal cancer after colonoscopy: a population-based, case-control study. Ann Intern Med 2011;154:22–30. 21200035 10.7326/0003-4819-154-1-201101040-00004

[8] Brandt HM , Dolinger HR, Sharpe PA, et al Relationship of colorectal cancer awareness and knowledge with colorectal cancer screening. Colorectal Cancer 2012;1:383–96. 26257828 10.2217/crc.12.45 PMC4529290

[9] American Cancer Society. Colorectal Cancer Early Detection, Diagnosis, and Staging. Available at: https://bit.ly/41h59I9 (12 April 2024, date last accessed).

[10] Qumseya BJ , Tayem YI, Dasa OY, et al Barriers to colorectal cancer screening in Palestine: a national study in a medically underserved population. Clin Gastroenterol Hepatol 2014;12:463–9. 24055899 10.1016/j.cgh.2013.08.051

[11] Centers for Disease Control and Prevention (CDC). Vital signs: colorectal cancer screening, incidence, and mortality—United States, 2002-2010. MMWR Morb Mortal Wkly Rep 2011;60:884–9. 21734636

[12] AlWaheidi S. Promoting cancer prevention and early diagnosis in the occupied Palestinian territory. J Cancer Policy 2023;35:100373. 36493987 10.1016/j.jcpo.2022.100373

[13] Elshami M , Ayyad M, Alser M, et al Awareness of colorectal cancer signs and symptoms: a national cross-sectional study from Palestine. BMC Public Health 2022;22:866. 35501803 10.1186/s12889-022-13285-8 PMC9063349

[14] Elshami M , Dwikat MF, Al-Slaibi I, et al Awareness of colorectal cancer risk factors in Palestine: where do we stand? JCO Glob Oncol 2022;8:e2200070. 35696626 10.1200/GO.22.00070 PMC9225594

[15] Elshami M , Naji SA, Dwikat MF, et al Myths and common misbeliefs about colorectal cancer causation in Palestine: a national cross-sectional study. JCO Glob Oncol 2024;10:e2300295. 38166235 10.1200/GO.23.00295 PMC10803036

[16] Elshami M , Ayyad M, Hamdan FK, et al Perceived barriers to early presentation and symptom-specific time to seek medical advice for possible colorectal cancer symptoms among Palestinians. Sci Rep 2023;13:6871. 37105988 10.1038/s41598-023-34136-5 PMC10140026

[17] Palestinian Central Bureau of Statistics. Palestine in Figures 2021. Available at: https://bit.ly/3i6G54C (12 April 2024, date last accessed).

[18] European Council on Foreign Relations. Mapping Palestinian Politics. Available at: https://bit.ly/3kEmO9c (12 April 2024, date last accessed).

[19] Power E , Simon A, Juszczyk D, et al Assessing awareness of colorectal cancer symptoms: measure development and results from a population survey in the UK. BMC Cancer 2011;11:366. 21859500 10.1186/1471-2407-11-366 PMC3188511

[20] Smith SG , Beard E, McGowan JA, et al Development of a tool to assess beliefs about mythical causes of cancer: the Cancer Awareness Measure Mythical Causes Scale. BMJ Open 2018;8:e022825. 10.1136/bmjopen-2018-022825 PMC6303629 30552257

[21] Simon AE , Juszczyk D, Smyth N, et al Knowledge of lung cancer symptoms and risk factors in the U.K.: development of a measure and results from a population-based survey. Thorax 2012;67:426–32. 22426791 10.1136/thoraxjnl-2011-200898

[22] Simon AE , Wardle J, Grimmett C, et al Ovarian and cervical cancer awareness: development of two validated measurement tools. J Fam Plann Reprod Health Care 2012;38:167–74. 21933805 10.1136/jfprhc-2011-100118 PMC3970720

[23] Abu Seir R , Kharroubi A, Ghannam I. Prevalence of tobacco use among young adults in Palestine. East Mediterr Health J 2020;26:75–84. 32043549 10.26719/2020.26.1.75

[24] Cancer Research UK. Can Eating Burnt Foods Cause Cancer? Available at: https://rb.gy/v66jo (12 April 2024, date last accessed).

[25] Harvard Humanitarian Initiative. KoBoToolbox. https://www.kobotoolbox.org (12 April 2024, date last accessed).

[26] Tseng T-S , Holt CL, Shipp M, et al Predictors of colorectal cancer knowledge and screening among church-attending African Americans and Whites in the Deep South. J Community Health 2009;34:90–7. 18941876 10.1007/s10900-008-9128-2

[27] Post D , Katz M, Tatum C, et al Determinants of colorectal cancer screening in primary care. J Cancer Educ 2008;23:241–7. 19058074 10.1080/08858190802189089 PMC4451163

[28] Power E , Van Jaarsveld CHM, McCaffery K, et al Understanding intentions and action in colorectal cancer screening. Ann Behav Med 2008;35:285–94. 18575946 10.1007/s12160-008-9034-y

[29] Al-Dahshan A , Chehab M, Bala M, et al Colorectal cancer awareness and its predictors among adults aged 50-74 years attending primary healthcare in the State of Qatar: a cross-sectional study. BMJ Open 2020;10:e035651. 10.1136/bmjopen-2019-035651 PMC7342467 32641359

[30] Schliemann D , Paramasivam D, Dahlui M, et al Change in public awareness of colorectal cancer symptoms following the Be Cancer Alert Campaign in the multi-ethnic population of Malaysia. BMC Cancer 2020;20:252. 32213173 10.1186/s12885-020-06742-3 PMC7093961

[31] Palestine Sports For Life: My Health My Wellbeing: 40 Day Campaign. Available at: https://bit.ly/3zAoDtB (12 April 2024, date last accessed).

[32] Mosli M , Alawadhi S, Hasan F, et al Incidence, prevalence, and clinical epidemiology of inflammatory bowel disease in the Arab World: a systematic review and meta-analysis. Inflamm Intest Dis 2021;6:123–31. 34722642 10.1159/000518003 PMC8527904

[33] Sindhu CK , Nijar AK, Leong PY, et al Awareness of colorectal cancer among the urban population in the Klang Valley. Malays Fam Physician off J Acad Fam Physicians Malays 2019;14:18–27. PMC7067497 32175037

[34] Knudsen MD , Hoff G, Tidemann-Andersen I, et al Public awareness and perceptions of colorectal cancer prevention: a cross-sectional survey. J Cancer Educ 2021;36:957–64. 32112366 10.1007/s13187-020-01721-5 PMC8520865

[35] McCaffery K , Wardle J, Waller J O Knowledge, attitudes, and behavioral intentions in relation to the early detection of colorectal cancer in the United Kingdom. Prev Med 2003;36:525–35. 12689797 10.1016/s0091-7435(03)00016-1

[36] Gimeno-García AZ , Quintero E, Nicolás-Pérez D, Jiménez-Sosa A. Public awareness of colorectal cancer and screening in a Spanish population. Public Health 2011;125:609–15. 21794885 10.1016/j.puhe.2011.03.014

[37] Elshami M , Albandak M, Alser M, et al. Screening Perspectives: The Role of Colorectal Cancer Awareness in Shaping Attitudes Toward Colonoscopy in Palestine. JCO Glob Oncol 2024;10:e2300470. 10.1200/GO.23.00470 PMC10898669 38386956

[38] Shahab L , McGowan JA, Waller J, Smith SG. Prevalence of beliefs about actual and mythical causes of cancer and their association with socio-demographic and health-related characteristics: findings from a cross-sectional survey in England. Eur J Cancer 2018;103:308–16. 29705530 10.1016/j.ejca.2018.03.029 PMC6202672

[39] Phelan SM , Griffin JM, Jackson GL, et al Stigma, perceived blame, self-blame, and depressive symptoms in men with colorectal cancer. Psychooncology 2013;22:65–73. 21954081 10.1002/pon.2048 PMC6000725

[40] Marlow LAV , Waller J, Wardle J. Does lung cancer attract greater stigma than other cancer types? Lung Cancer 2015;88:104–7. 25704958 10.1016/j.lungcan.2015.01.024

