# Supplementary material for: Differences in colorectal cancer awareness between screening eligible vs. ineligible Palestinians: a national cross-sectional study
Source: Eur J Public Health. 2024 May 14;34(5):872–8. doi: 10.1093/eurpub/ckae083 (PMC11430923; doi:10.1093/eurpub/ckae083)
Supplement: ckae083_Supplementary_Data [file ckae083_supplementary_data.pdf]

**Supplementary table 1:** Characteristics of study participants.			
**Characteristic**	**Screening-ineligible (n= 2158)**	**Screening-eligible (n= 540)**	**p–value**
**Age**, median [IQR]	27.0 [23.0, 34.0]	52.0 [47.0, 57.0]	<0.001
**Gende**r, n (%)			
Male	966 (44.8)	257 (47.6)	0.24
Female	1192 (55.2)	283 (52.4)	
**Educational level**, n (%)			
Secondary or below	817 (37.9)	371 (68.7)	<0.001
Post–secondary	1341 (62.1)	169 (31.3)	
**Occupation**, n (%)			
Unemployed/housewife	854 (39.6)	264 (48.9)	<0.001
Employed	939 (43.5)	230 (42.6)	
Retired	5 (0.2)	46 (8.5)	
Student	360 (16.7)	0 (0.0)	
**Monthly income ≥ 1450 NIS**, n (%)	1422 (65.9)	382 (70.7)	0.032
**Marital status**, n (%)			
Single	892 (41.3)	12 (2.2)	<0.001
Married	1241 (57.5)	483 (89.4)	
Divorced/Widowed	25 (1.2)	45 (8.3)	
**Residency,** n (%)			
Gaza Strip	923 (42.8)	187 (34.6)	<0.001
West Bank and Jerusalem	1235 (57.2)	353 (65.4)	
**Having a chronic disease**, n (%)	186 (8.6)	272 (50.4)	<0.001
**Knowing someone with cancer**, n (%)	387 (17.9)	127 (23.5)	0.003
**Site of data collection**, n (%)			
Public Spaces	741 (34.3)	114 (21.1)	<0.001
Hospitals	770 (35.7)	264 (48.9)	
Primary healthcare centers	647 (30.0)	162 (30.0)	
n= number of participants, IQR= interquartile range.			
